# Influence of the Carbon and Nitrogen Sources on Diabolican Production by the Marine *Vibrio diabolicus* Strain CNCM I-1629

**DOI:** 10.3390/polym14101994

**Published:** 2022-05-13

**Authors:** Christine Delbarre-Ladrat, Corinne Sinquin, Laetitia Marchand, Sandrine Bonnetot, Agata Zykwinska, Véronique Verrez-Bagnis, Sylvia Colliec-Jouault

**Affiliations:** Ifremer, MASAE, EM3B Laboratory, CEDEX 3, F-44300 Nantes, France; corinne.sinquin@ifremer.fr (C.S.); laetitia.marchand@ifremer.fr (L.M.); sandrine.bonnetot@ifremer.fr (S.B.); agata.zykwinska@ifremer.fr (A.Z.); veronique.verrez@ifremer.fr (V.V.-B.); sylvia.colliec.jouault@ifremer.fr (S.C.-J.)

**Keywords:** exopolysaccharide, marine bacterium, production, yield, central composite design, molecular weight

## Abstract

Recent advances in glycobiotechnology show that bacterial exopolysaccharides (EPS) presenting glycosaminoglycan (GAG)-like properties can provide a valuable source of bio-active macromolecules for industrial applications. The HE800 EPS, named diabolican, is a marine-derived anionic high-molecular-weight polysaccharide produced by *Vibrio diabolicus* CNCM I-1629 which displays original structural features close to those of hyaluronic acid. We investigated the impact of carbon and nitrogen substrates on both *Vibrio diabolicus* growth and diabolican production. Both substrates were screened by a one-factor-at-a-time method, and experimental designs were used to study the effect of glucose, mannitol, and ammonium acetate various concentrations. Results showed that the medium composition affected not only the bacterium growth and EPS yield, but also the EPS molecular weight (MW). EPS yields of 563 and 330 mg L^−1^ were obtained in the presence of 69.3 g L^−1^ glucose and 24.6 g L^−1^ mannitol, respectively, both for 116.6 mM ammonium acetate. MW was the highest, with 69.3 g L^−1^ glucose and 101.9 mM ammonium acetate (2.3 × 10^6^ g mol^−1^). In parallel, the bacterial maximum specific growth rate was higher when both carbon and nitrogen substrate concentrations were low. This work paves the way for the optimization of marine exopolysaccharide production of great interest in the fields of human health and cosmetics.

## 1. Introduction

Glycosaminoglycans (GAGs) are ubiquitous glycopolymers found in animal tissues and are essential for development and organogenesis. In particular, GAGs have been shown to participate in many biological processes such as cell adhesion, migration, proliferation, and differentiation through interactions with proteins, such as growth factors, proteases, and chemokines [1,2]. Therapeutic GAGs are conventionally extracted from animal tissues; however, the marketing of products of animal origin is more and more restricted because of health hazards due to potential viral contamination. Consequently, efforts have been made to replace animal-derived GAGs with non-mammalian GAG-mimetics produced by microorganisms such as bacteria [2].

Diabolican (HE800 EPS name built with “diabolic” from the producing bacterial species name, and “an” referring to glycan) is produced by the non-pathogenic marine *Vibrio diabolicus* strain CNCM I-1629 [3]. It is constituted of tetrasaccharide repeating units with the structure →3)-β-D-GlcNAc-(1→4)-β-D-GlcA-(1→4)-β-D-GlcA-(1→4)-α-D-GalNAc-(1→ [4]. GlcA residue confers a polyanionic nature to the EPS. Diabolican has been shown to promote bone reconstruction and improve in vitro skin regeneration [5,6]. Both structural and biological features of this EPS are suitable for obtaining a lead drug candidate offering an alternative to GAGs from animal origin in the treatment of different cellular disorders observed in various diseases.

Diabolican and other GAG-mimetic polysaccharides hold great economic value due to their versatile biological activities, mainly in the biomedical and cosmetic fields [7,8,9]. Thanks to efficient fermentation technologies, the cultivation of bacterial strains can provide a safer source of GAG-like polysaccharides. However, the industrial production of marine bacterial EPS requires higher yields, lower production costs, and scale-up robustness. The HE800 EPS, usually obtained by lab-scale fermentation at 0.5 g L^−1^, presents a low yield compared to other industrial bacterial polysaccharides. For example, xanthan or hyaluronic acid are produced by *Xanthomonas* or wild type *Streptococcus* bacteria at a scale above 10 g L^−1^ [10,11].

Polysaccharide synthesis and yield depend on environmental and nutritional conditions [12]. A large number of reports address the modulatory effect of various cultivation parameters on the yield of bacterial EPS production, including agitation and dissolved oxygen, temperature, pH, and nutrient concentrations such as carbon and nitrogen sources [13,14,15,16,17,18]. Nitrogen substrates are essential nutrients for cell growth and protein biosynthesis in all organisms, while carbon compounds provide carbon and energy to cellular metabolic pathways. Therefore, medium optimization is required to achieve economically significant production of valuable EPS [19]. The design of experiments (DoE) including Plackett–Burman (PB) design for the screening of factors, central composite design (CCD) and response surface methodology (RSM), allows growth and production medium optimization with limited cost and time by involving statistical methods [20,21]. On the other hand, high molecular weight (HMW) is a highly desirable characteristic for applying EPS in biomedical such as ophthalmology, orthopedics, wound healing, and cosmetic fields, since biological roles are dependent upon molecular weight (MW) [8,22].

In this work, we explored the effect of carbon and nitrogen substrates on the production yield of diabolican EPS through *V. diabolicus* fermentation. As a first step, one-single-factor-at-a-time experiments allowed the selection of the best substrates to combine. CCD and RSM were subsequently used to determine the optimal conditions for maximal EPS production. They were also used to estimate whether carbon and nitrogen type and concentrations can contribute to EPS MW mitigation.

## 2. Materials and Methods

Unless otherwise stated, all chemicals were purchased from Sigma Aldrich Chimie (Saint-Quentin-Fallavier, France).

### 2.1. Microbiology

*Vibrio diabolicus* strain CNCM I-1629 was isolated from a sample collected in October 1991 at a deepsea hydrothermal vent site from the East Pacific ridge [3]. It was stored in microtubes at −80 °C with 20% (*v*/*v*) glycerol. This bacterial strain is usually grown on Zobell medium (Z) composed of 33.3 g L^−1^ aquarium salts (Instant Ocean), 4 g L^−1^ tryptone (Organotechnie, La Courneuve, France), and 1 g L^−1^ Bacto yeast extract (Biokar diagnostics, Beauvais, France). Glucose at 30 g L^−1^ is added to obtain production of the diabolican EPS. The initial pH of the medium is 7.2. All cultures, either in microplates or 2 L baffled Erlenmeyer flasks, were seeded at 1/50 (*v*/*v*) with the inoculum prepared either in the final medium or diluted in salted water, and incubated at 30 °C and 150 rpm. Seeding ratio was 1/10 in the bioreactor.

### 2.2. Selection of a New Medium

For this study, a new buffered and chemically defined medium was needed. Phosphate buffer (15.3 g L^−1^ K_2_HPO_4_ and 1.6 g L^−1^ KH_2_PO_4_) was used. Moreover, aquarium salts were replaced by 20 g L^−1^ NaCl to avoid formation of white precipitate. A low amount of yeast extract (0.5 g L^−1^) was added to provide essential oligo-elements. This basic solution was prepared 4 times, concentrated. Various sources of nitrogen and carbon were added for bacterial growth and EPS production. Those carbon and nitrogen substrates were also prepared 4 times, concentrated. The final medium, named S1, was obtained by mixing separately autoclaved components as follows: 1 volume of 4× basic solution, 1 volume of 4× carbon substrate solution, 1 volume of 4× nitrogen source solution, 1 volume of water, 2 mL L^−1^ of 1 M MgSO_4_ 2H_2_O, and 2 mL L^−1^ of trace elements (2 mg L^−1^ CaCl_2_ 2H_2_O, 2 mg L^−1^ FeSO_4_ 7H_2_O, 0.1 mg L^−1^ H_3_BO_3_, 0.4 mg L^−1^ CuSO_4_ 6H_2_O, 6 mg L^−1^ MnCl_2_ 4H_2_O, 2 mg L^−1^ ZnCl_2_, 0.1 mg L^−1^ Na_2_MoO_4_ 2H_2_O, 2 mg L^−1^ FeCl_3_ 6H_2_O).

Preliminary studies to evaluate this medium were performed in microplates and maximum specific growth rate was measured as indicated below. The EPS production was evaluated with agarose gel electrophoresis. Cultures were centrifuged at 15,000 rpm, 30 min, 10 °C. Twenty microliters of the supernatants of the fourth microplate line (8-fold dilution) were loaded onto the agarose gel. Electrophoresis and staining were carried out as previously described [23].

### 2.3. Determination of Growth Rate

Maximum specific growth rate (µmax) was determined at 620 nm in 96-well microplates with Varioskan microplate reader (Thermo Fisher Scientific, Illkirch, France). Since optical density (OD) varies with cell morphology which can change upon growth, especially in the presence of carbon substrate, e.g., glucose, we used a series of half-dilutions [24]. Each well of the microplate was filled with 200 µL of the appropriate medium. Each column contained the same medium. The inoculum was prepared in Zobell medium and diluted 100× in salted water before loading on the first line of the microplate (200 µL in each well). Two hundred microliters of the first well of the considered column were sampled and transferred into the second well below. This step was repeated until the second last well of the microplate column. Then, 200 µL of this second last well were discarded. The last well of each column received no inoculation for control. Finally, each well contained 200 µL of a mixture medium and in-series diluted inoculum. Microplates were incubated at 30 °C under medium shaking force of the orbital movement at 300 rpm. Growth was monitored by measuring OD at 620 nm every 15 min. For each condition (one microplate column), 9 OD curves were registered. The detection times for three OD values (0.5, 0.6 and 0.7 chosen in the middle of the exponential growth phase) were estimated and plotted against the neperian logarithm of the dilution fold with a script developed in R. The maximum specific growth rate (µmax) was calculated as the opposite of the slope of the linear regression [25]. Standard deviation was estimated with experimental triplicates for each medium and three OD values per serial dilutions. Data were analyzed for significance by the Student’s t-test. The significance threshold was set to 0.05.

Growth in Erlenmeyer flasks was followed by measuring OD at 620 nm of withdrawn samples with cuvettes and a UV-visible spectrophotometer (UV-1800, Shimadzu, Marne-la-Vallée, France).

### 2.4. Carbon and Nitrogen Sources Studied and Central Composite Design (CCD)

Experiments to study the effect of various carbon and nitrogen sources, as well as the CCD were conducted in baffled 2 L Erlenmeyer flasks containing 400 mL of broth medium. Cultivation was carried out at 30 °C under 150 rpm shaking.

Optimization experiments were designed according to CCD with two factors, including central and axial points. These parameters varied symmetrically around the central point according to the factorial design (Table 1). CCD MW and yield results were analyzed with Statgraphics Centurion XVII version 17.2.07 based on ANOVA. Ammonium acetate was the nitrogen substrate, and glucose or mannitol was the carbon source. The significance threshold was set to 0.05.

### 2.5. Cultivation in Bioreactor

EPS production was performed in 48 h batch cultures in a 2 L bioreactor (BioFlo 110, New Brunswick Scientific, Paris, France) containing 1 L of S1 cultivation medium with ammonium acetate appropriate concentration. A concentrated solution of glucose was sterilized separately and added to start the culture. Batches were performed under controlled conditions: temperature (30 °C), agitation 300 rpm, fixed aeration rate (0.5 *v*/*v*/*m*) and a regulated pH of 7.2 by automated addition of 1 M NaOH. Bacterial growth was monitored by measuring the turbidity at 620 nm of the culture diluted 5× in 20 g L^−1^ NaCl (UV-Visible spectrophotometer UV-1800, Shimadzu, Marne-la-Vallée, France). Foaming was avoided by adding pluronic acid oil (BASF, Sigma Aldrich Chimie, Saint-Quentin-Fallavier, France).

### 2.6. EPS Recovery and Characterization

Cultures in Erlenmeyer flasks or bioreactors were centrifuged at 8000× g for 30 min at 10 °C (Avanti J-E, Beckman Coulter, Roissy CDG, France). Pellet was discarded. The supernatant was filtered under vacuum on 2.6 µm and 0.7 µm glass microfiber membranes (Whatman, Thermo Fisher Scientific, Illkirch, France), and further purified by tangential ultrafiltration with a membrane of 100 kDa cut-off (Pellicon 2, Millipore, VWR, Rosny-sous-Bois, France) until the conductivity was close to water. Purified EPS were freeze-dried.

Total carbohydrate was estimated by the modified phenol-sulfuric acid method of Dubois, et al. (1956), using glucose as standard [26].

Monosaccharide composition was determined according to Kamerling et al. method [27], modified by Montreuil, et al. [28]. Samples were hydrolyzed for 4 h at 100 °C by 3 M MeOH/HCl with myo-inositol used as the internal standard. After re-*N*-acetylation with acetic anhydride overnight at room temperature, the methyl glycosides were converted into their corresponding trimethylsilyl derivatives using *N*,*O*-bis(trimethylsilyl)trifluoroacetamide and trimethylchlorosilane (BSTFA:TMCS) 99:1 (Merck). Separation and quantification of the per-*O*-trimethylsilyl methyl glycosides were performed by gas chromatography (GC-FID, Agilent Technologies 6890N). Monosaccharide content (wt/wt. %) was determined in triplicates.

Protein content was analyzed using the bicinchoninic acid method [29] (BCA Protein Assay Kit, Sigma Aldrich Chimie, Saint-Quentin-Fallavier, France).

Weight–average molecular weight was determined using High-Performance Size-Exclusion Chromatog-raphy (HPSEC, Prominence Shimadzu, Marne-la-Vallée, France) coupled with a multiangle light scattering (MALS, Dawn Heleos-II, Wyatt Technology, Toulouse, France) and a differential refractive index (RI) (Optilab, Wyatt Technology, Toulouse, France) detectors. HPSEC system was composed of a HPLC system Prominence Shimadzu, a PL aquagel-OH mixed, 8 μm (Agilent) guard column (U 7.5 mm × L 50 mm), and a PL aquagel-OH mixed (Agilent) separation column. Samples were eluted with 100 mM ammonium acetate. The molecular weight was calculated using a refractive index increment characteristic of polysaccharides, dn/dc = 0.145 mL g^−1^.

Production yield was calculated from the recovered amount of the lyophilizate. This amount was corrected in consideration of the proportion of carbohydrate amount in the lyophilized sample, as indicated by the recovery percentage in the molecular weight analysis report (RI).

## 3. Results

### 3.1. Selection of a Buffered Chemically Defined Medium

**Figure 1:**
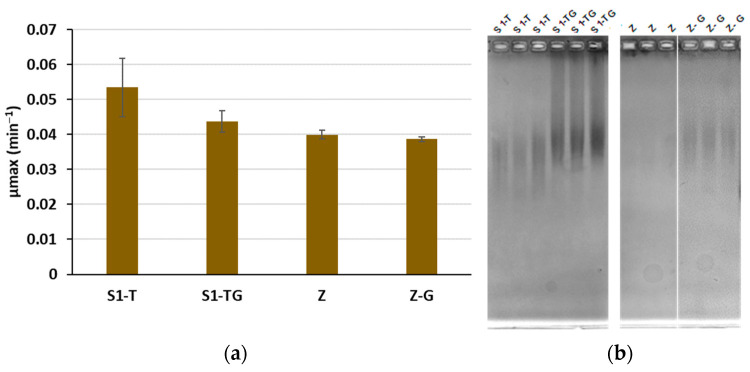
Comparison of the growth of *V. diabolicus* (**a**) and of HE800 EPS production (**b**) on Zobell medium (Z) and S1 medium with tryptone (S1-T), supplemented or not with 30 g L^−1^ glucose (G). Standard deviation of µmax was estimated with triplicates.

Marine bacterial EPS are usually produced at lab scale on Zobell medium supplemented with glucose in bioreactors. However, when testing a large number of cultivation conditions, e.g., 11 for the CCD, it is not possible to carry out the experiments in bioreactors. Therefore, we used Erlenmeyer flasks to study the influence of carbon and nitrogen substrates on EPS production. A buffered chemically defined medium was needed. For this purpose, the S1 medium was prepared and evaluated in microplates. To compare it with Zobell medium (Z), the S1 medium was supplemented with 4 g L^−1^ tryptone. The addition of 30 g L^−1^ glucose, necessary for the EPS production, was also tested. No significant difference in the maximum specific growth rate (µmax) was observed between S1-Tryptone (S1-T) and Z, nor between the media supplemented with 30 g L^−1^ glucose (S1-TG and Z-G) on the other hand (*p*-value > 0.05) (Figure 1a). The agarose gel electrophoresis showed that the addition of glucose in both culture media (Z-G and S1-TG) is required to produce the EPS. S1-TG seemed to increase both the EPS amount and MW compared to Z-G (Figure 1b). The production in a bioreactor (S1-TG medium, 30 °C, pH 7.2, 25 L h^−1^ aeration, 250 rpm agitation, 48 h duration) led to the EPS recovery at a yield of 433 mg L^−1^ and a MW of 2.4 ± 0.34 × 10^6^ g mol^−1^, which validated the S1-TG medium for the EPS production.

### 3.2. Selection of Carbon and Nitrogen Sources

**Figure 2:**
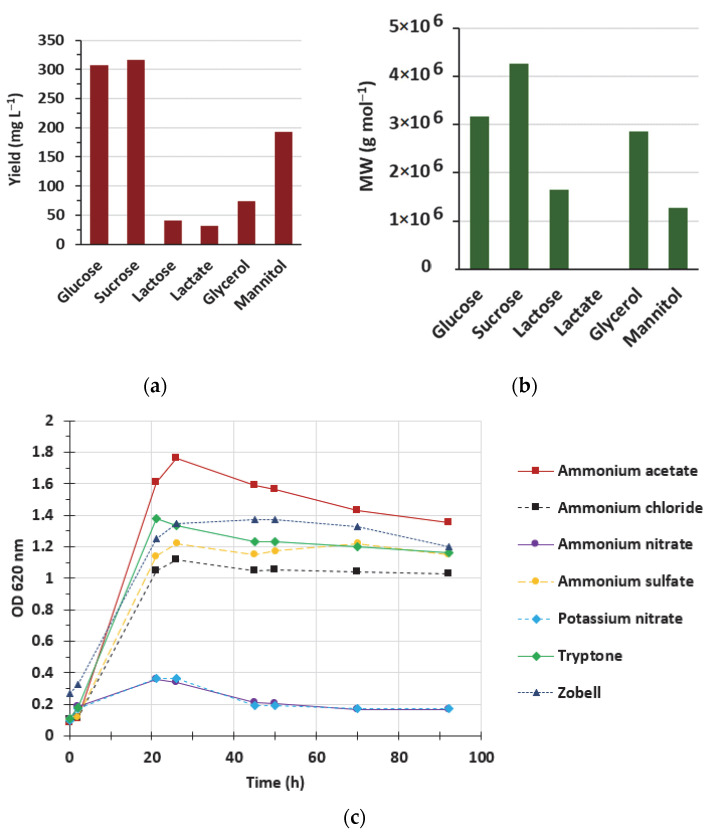
Screening of carbon sources on EPS production yield and MW (**a**,**b**) and nitrogen sources on bacterial growth (**c**). (**a**,**b**) were carried out with 100 mM ammonium chloride; (**c**) was studied with 30 g L^−1^ glucose. All cultures were performed in 2 L baffled Erlenmeyer flasks containing 400 mL of medium.

Preliminary experiments were carried out to determine suitable carbon and nitrogen sources for both bacterial growth and EPS production. All these experiments were carried out in 400 mL medium broth with 2 L baffled Erlenmeyer flasks. Firstly, we tested the impact of various carbon sources on EPS yield and MW (Figure 2a,b). Ammonium chloride (100 mM) was chosen as the nitrogen source according to other studies [30,31,32]. Various carbon sources were used: glucose, sucrose, lactose, lactate, glycerol, and mannitol at 30 g L^−1^. Diabolican was produced with all the carbon sources except lactose and lactate. The best yields were obtained with glucose (307 mg L^−1^) and sucrose (316 mg L^−1^); low residual substrate in the EPS molecule (2 and 4 wt. % of pure EPS, respectively) was detected. The yield obtained on mannitol was lower but acceptable (193 mg L^−1^) and only 0.5 wt. % of residual mannitol was detected in the EPS. The highest MW was obtained when the EPS was produced with sucrose or glucose (4.3 × 10^6^ g mol^−1^ and 3.1 × 10^6^ g mol^−1^, respectively); MW with glycerol was slightly lower (2.9 × 10^6^ g mol^−1^). To investigate further the substrate’s impact on EPS production and its MW, we selected glucose, the usual sugar carbon source, and mannitol, because of its low residual content in the final product. In addition, for this polyol, a metabolic pathway could be distinct from glucose, having a different effect on the EPS produced.

Different salts including ammonium sulfate, ammonium chloride, potassium nitrate, ammonium acetate, and ammonium nitrate at 100 mM, as well as tryptone at 4 g L^−1^ were tested as inorganic or organic nitrogen sources for bacterial growth. 30 g L^−1^ glucose was added to all media (Figure 2c). Zobell medium was used as a control. None of the nitrate salts allowed any significant growth. The best growth (growth rate and maximal optical density) was obtained with ammonium acetate, while ammonium chloride induced the lowest bacterial growth. The growth observed on the S1 medium containing either ammonium sulfate or tryptone, and on the Zobell medium were comparable.

Based on these results, both ammonium acetate and ammonium sulfate were selected for the next experiment to test their effect on the EPS production together with glucose and mannitol as carbon sources.

**Figure 3:**
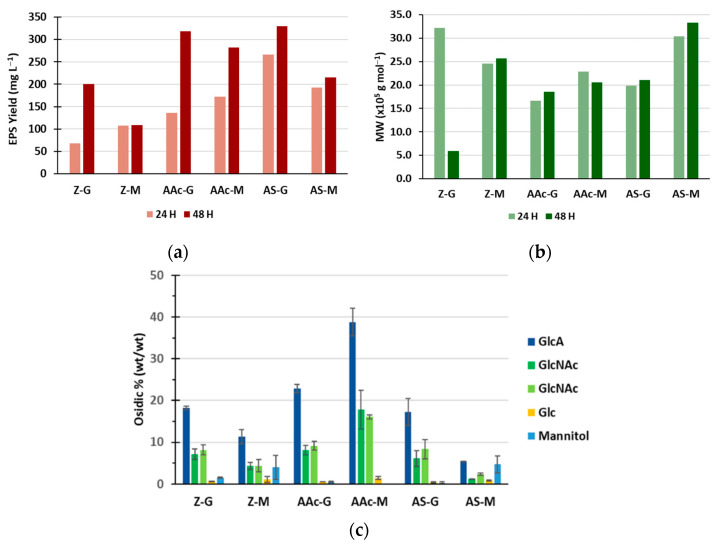
Features of the EPS produced on various media: EPS yield at 24 and 48 h (**a**), MW at 24 and 48 h (**b**), and osidic composition at 48 h (**c**). Media: Z-G: Zobell Glucose, Z-M: Zobell Mannitol, AAc-G: Ammonium acetate Glucose, AAc-M: Ammonium acetate Mannitol, AS-G: Ammonium sulfate Glucose, AS-M: Ammonium Acetate Mannitol. Oses: Glucuronic acid (GlcA), *N*-Acetyl glucosamine (GlcNAc), *N*-Acetyl galactosamine (GalNAc), Glucose (Glc). Samples of culture broth (200 mL) were withdrawn at 24 h and 48 h. Osidic percentages are mean ± standard deviations for triplicate analyses.

Figure 3a shows that the highest EPS yields were achieved after a 48h-culture on two glucose supplemented media containing ammonium acetate or ammonium sulfate as the nitrogen source (AAc-G and AS-G). The production yield in ammonium acetate and mannitol (AAc-M) was nearly as high as these two glucose-supplemented media (350 mg L^−1^). In addition, the production yield in Zobell supplemented with glucose (Z-G) was lower than with ammonium salts. Mannitol was a slightly less efficient carbon source than glucose for diabolican production, confirming results observed in Figure 2a. Molecular weight varied in the range of 5.9 × 10^5^–3.3 × 10^6^ g mol^−1^ (Figure 3b). MW observed at 24 h and 48 h of culture were similar, except for Z-G. The important decrease of the MW at 48 h in Z-G might be due to the EPS hydrolysis due to medium acidification at the end of fermentation or enzymatic degradation as it was already observed for other bacterial strains [33,34]; this decrease was neither observed with mannitol nor in S1-based media (AAc-G, AAc-M, AS-G, AS-M).

The monosaccharide composition of the EPS at 48 h did not vary with medium composition (Figure 3c) and was consistent with the structure of the repeating unit described by Rougeaux, et al. [4]. The overall osidic composition amount was higher in media containing ammonium acetate (representing 40.2% for AAc-G and 72.7% for AAc-M of the lyophilisate). This proportion was also higher when mannitol was used as the carbon source.

Taken together, these results allowed the selection of media consisting of ammonium acetate as the nitrogen source and glucose or mannitol as the carbon source for further experiments.

### 3.3. Effects of Carbon and Nitrogen Sources on Growth and EPS Production

**Figure 4:**
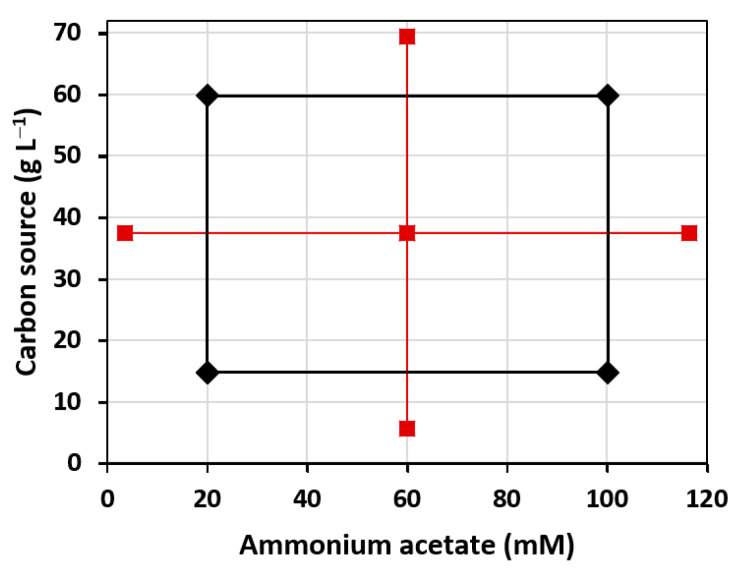
Representation of the CCD used in this study. Black diamonds: cube points; Red squares: central point and star points.

The combined effects of ammonium acetate with either glucose or mannitol on the growth rate of *V. diabolicus*, production yield and molecular weight of the EPS were analyzed using a Design of Experiment (DoE) approach through a central composite design (CCD) and response surface methodology (RSM) analysis. The carbon source domain was 5.68–69.3 g L^−1^, with a central point at 37.5 g L^−1^. Ammonium acetate concentrations, centered at 60 mM, varied from 3.43 mM to 116.57 mM, according to the literature [30,31,32] (Figure 4). The other conditions were set from those routinely used for the production of EPS in the laboratory (temperature 30 °C, 48 h cultivation, 150 rpm shaking).

*V. diabolicus* growth rate was studied in microplates as a function of carbon and nitrogen sources using the central composite design while EPS production was carried out in baffled Erlenmeyer flasks to obtain enough EPS to perform the analyses. In this way, all 11 experiments of the CCD could be performed at once.

The molar ratio of 2:1:1 for GlcA:GlcNAc:GalNAc composition of the EPS was conserved in all media, demonstrating that the concentrations of carbon and nitrogen regardless of their sources had no major effect on the polysaccharide chemical composition and structure (Supplementary Data S1). In particular, EPSs produced upon mannitol had the same composition as upon glucose, as already observed in Figure 2c. The overall osidic composition yield varied on the media.

**Figure 5:**
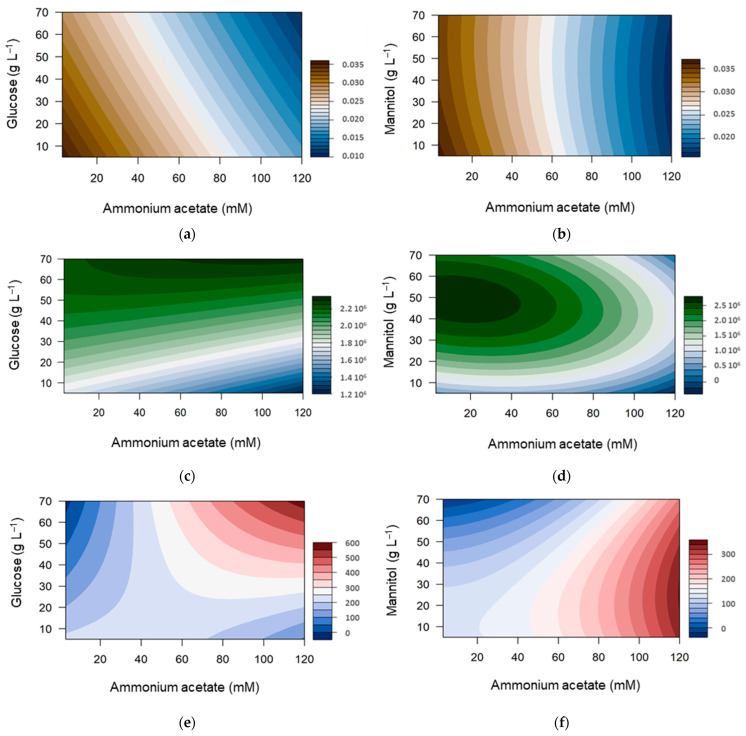
Contour plots of the combined effect of carbon and nitrogen sources on maximum growth rate (**a**,**b**), molecular weight (**c**,**d**), and production yield (**e**,**f**) as predicted by the model determined with Statgraphics. Cultivation was carried out on ammonium acetate and glucose (**a**,**c**,**e**) or mannitol (**b**,**d**,**f**).

All data were analyzed with Statgraphics software with a quadratic model including the main effect of each factor, cross-interaction between them and second-order term for each of the variables. The CCD responses were statistically analyzed, and the generated models were used to draw the response surfaces (Figure 5). Statistical attributes of the models are reported in Supplementary Data S2. Residual plots all appear random, thus indicating that the residuals may be independent of each other. Similarly, the Durbin–Watson tests together with the lag 1 residual autocorrelation indicate no autocorrelation among residuals at the 5% confidence level (*p*-value > 0.05). Then, R-squared and other statistics could be assessed. The correlation coefficients (R-squared) used to determine the relationship between the experimental and the predicted responses reached 99% for µmax on both carbon sources, indicating that the model explained the µmax variability very well. Yield R-squared coefficients (90% and 70% on glucose and mannitol, respectively) and MW ones (around 65% for both carbon sources) were weaker. Purification steps, including centrifugation, ultrafiltration, and freeze-drying, are required before yield and MW determination. These steps are all likely to increase the pure error.

Ammonium acetate contributed significantly to the µmax fitted models (*p*-value < 0.05), in the presence of all other variables. Both carbon sources had distinct behaviors, since the glucose effect on µmax was significant, while the mannitol one was not. MW was significantly influenced by glucose but not by mannitol. Finally, ammonium acetate, glucose, and their interaction significantly influenced the yield. Meanwhile, only the effect of ammonium acetate on EPS yield was significant in the mannitol RSM.

**Figure 6:**
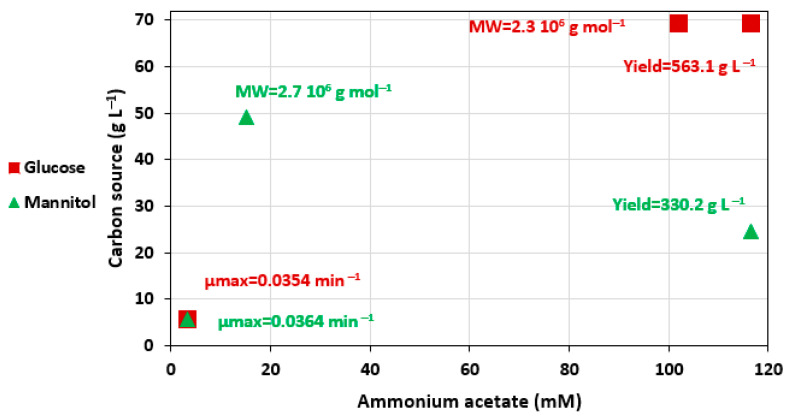
Optima determined by the fitted models for µmax, EPS MW and yield for both ammonium acetate/glucose or ammonium acetate/mannitol CCDs.

Optima given by these models are shown in Figure 6. The maximal specific growth rate is maximum at the lower boundaries of the domain. In contrast, MW and yield were optimal at the higher boundaries corresponding to the maximal glucose and ammonium acetate concentrations. The mannitol RSM indicated that ammonium acetate and mannitol should be, respectively, high and medium for maximal yield, while they should be, respectively, low and medium for higher MW. Optimal MW were similar for both carbon sources, but the optimal yield was much higher with glucose than with mannitol, although both substrates provided virtually the same carbon input.

**Figure 7:**
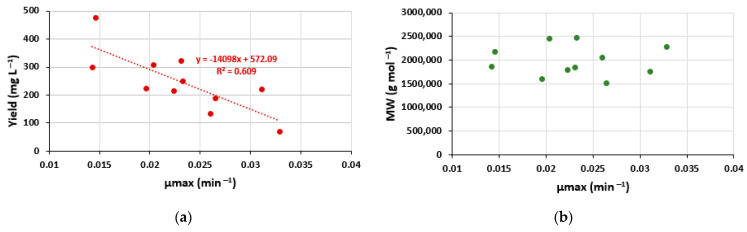

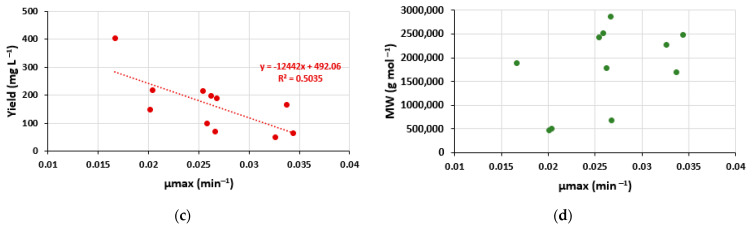
Relationship between yield or MW and growth rate. Points are experimental values in the CCDs with glucose (**a**,**b**) and mannitol (**c**,**d**).

The growth rate decreased when glucose was increased. In contrast, MW and yield were higher with high glucose concentrations. This result suggests that high glucose and ammonium acetate concentrations stressed the bacteria cells; however, they remained necessary for the EPS production. In addition, a decreasing tendency between the yield and µmax was observed (Figure 7), confirming that the best yields were obtained when the bacterial strain grew more slowly.

All these data suggest that carbon and nitrogen substrate concentrations can modulate both MW and yield. This is an important point for the industrialization of the production process and for obtaining an EPS with a targeted MW with optimized efficiency.

### 3.4. Batch Fermentation in Bioreactor

**Figure 8:**
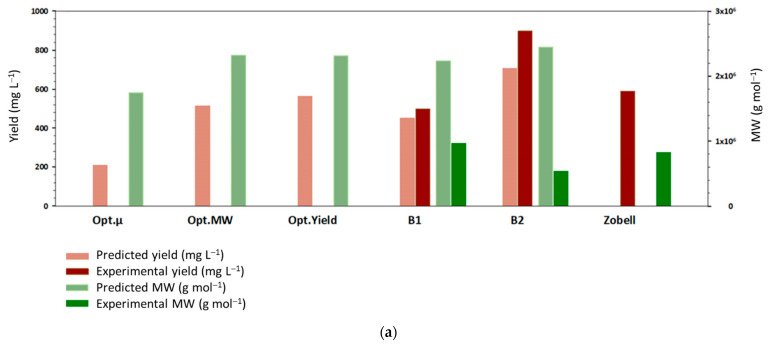

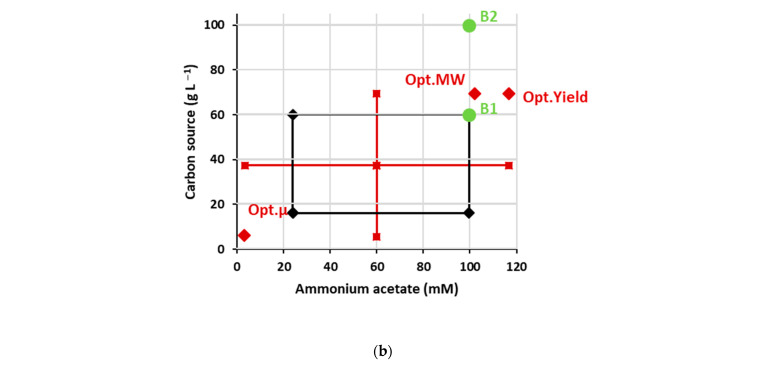
Graphical representation of experimental and predicted MW and yield values (**a**) and schematic representation of the ammonium acetate and glucose concentration combinations (**b**). B1, B2, and Zobell experimental points were obtained in bioreactors. MW and yield were predicted with the models at ammonium acetate and glucose concentrations optimal for growth rate (Opt.µ), MW (Opt.MW), and yield (Opt.Yield).

CCD experiments were carried out in Erlenmeyer bottles to be able to perform all of them at the same time; it was thus interesting to compare and validate the results with cultures in a bioreactor. Glucose was chosen as the carbon source. One experimental point was positioned at the upper limits of the domain (B1), near the MW and the yield optima determined by the RSM; another one (B2) was positioned outside the CCD domain. Productions in conventional conditions (Zobell medium supplemented by 30 g L^−1^ of glucose) were also performed (Table 2 and Figure 8).

EPS yields and MW at Opt.MW and Opt.Yield substrate concentrations were comparable with each other, showing that the highest substrate concentrations led to both the best yield and the highest MW. The values of yield and MW at Opt.µ demonstrated that both ammonium acetate and glucose concentrations were optimal for growth and sub-optimal for EPS production, suggesting that growth and EPS production could be competing pathways in the bacterial strain (Figure 8).

Both MW and yield obtained with Zobell supplemented with 30 g L^−1^ glucose were close to those obtained previously in the lab. Interestingly, the predicted yield values for B1 and B2 established in shake flasks were consistent with the experimental yields obtained in the bioreactor. The highest yield was achieved for B2 both experimentally and by model extrapolation. This suggests that an increase in glucose and ammonium acetate could result in a further increase in the production yield. MW obtained experimentally in the bioreactor was lower than the predicted one in the Erlenmeyer flasks. In addition, for B2, MW was also lower than the one obtained for B1 indicating that in the bioreactor, MW decreased when glucose concentration further increased outside the CCD domain. We suggest that optimal ammonium acetate and glucose concentrations for the highest MW were located between B1 and B2 values. Overall, even if some discrepancies were observed between cultures performed in the Erlenmeyer flask or in the bioreactor, cultures in baffled Erlenmeyer flasks remained an efficient method to optimize the EPS production.

## 4. Discussion

The optimization of the marine exopolysaccharides production is of great interest and is a prerequisite for their industrial development for further applications. The biological properties of the diabolican offer a promising alternative to GAGs from animal origin, which are mainly used at present in the fields of human health and cosmetics. The production of EPS or other bacterial products is widely optimized using DoE [20]. However, studies on the impact of substrate concentrations on MW values are rare. In this study, we used RSM to optimize the fermentation conditions of *V. diabolicus* CNCM I-1629 in order to improve its EPS production. First, we screened several carbon and nitrogen sources and found that the optimal carbon source was glucose, a carbon substrate commonly used for EPS production by bacteria [35]. Mannitol was also investigated as a polyol carbon source. We also screened inorganic nitrogen sources. The use of an inorganic nitrogen source has great advantages over peptones (protein hydrolysates with complex composition), as it makes it possible to identify each chemical element present in the culture medium, as well as its concentration; such a chemically defined medium is a tangible tool for accurate metabolic studies.

The highest yields of EPS, 563 and 330 mg L^−1^, were obtained with 116.6 mM ammonium acetate and 69.3 g L^−1^ glucose or 24.6 g L^−1^ mannitol, respectively. The highest MW was 2.3 × 10^6^ g mol^−1^ and was obtained with 101.9 mM ammonium acetate and 69.3 g L^−1^ glucose. The highest glucose concentration gave the highest yield. Indeed, growth and EPS production require carbon for energy supply [21]. Nevertheless, since optima were near the CCD domain boundaries, higher nitrogen and carbon source concentrations should be investigated to further dissect the EPS biosynthesis mechanism. In our study, the maximum specific growth rate and EPS production yield could be accurately predicted by adjusting ammonium acetate and glucose or mannitol concentrations. However, the correlation coefficients of the MW models were relatively low (around 60%), suggesting an unidentified factor effect during EPS biosynthesis.

Substantial evidence on the influence of medium composition on the amount and MW of EPS produced by bacteria has been accumulated. The yield of hyaluronic acid produced by *Streptococcus zooepidemicus* was enhanced with 20% dissolved oxygen and a 10:20 ratio of glucose: *N*-acetyl glucosamine resulted in the highest MW compared to glucose as the sole carbon source [36]. Oleksy-Sobczak et al. reported that the main medium component determinant for the EPS synthesis by *Lactobacillus rhamnosus* strains is the carbon source, with the optimal ones being fructose and sucrose [18]. Interestingly, lactose was the best substrate for EPS production by *Alteromonas macleodii* thanks to its β-galactosidase enzyme; 18.7 g L^−1^ EPS were obtained in 72 h with 50 g L^−1^ lactose [37]. Growth medium composition not only influences the yield of bacterial EPS, but might also have an impact on the structural features such as MW and the chemical composition. To the best of our knowledge, there is no clear indication that the medium composition could mitigate the final EPS chemical structure; most likely, the observed variation might be due to different EPS with differentially regulated production [12,38]. Bejar et al. reported that *Volcaniella eurihalina* produced two different EPS in two different culture media [39]. In this study, the osidic composition of diabolican was the same whatever the growth medium used.

Polyols such as glycerol have already been studied as a carbon source in the production of EPS by *Yangia* sp., but it was not as efficient as maltose, fructose and sucrose [32]. Glucose and glycerol were the best carbon substrates for the EPS production by *Pseudoalteromonas* sp. AM with organic nitrogen sources, with inorganic ones supporting only weakly the bacterial growth and the EPS production [40]. Ates et al. showed that mannitol enhanced the levan production by levansucrase in *Halomonas smyrnensis* in the presence of glucose, by changing glucose and fructose fluxes [41]. Mannitol is accumulated in some living organisms, especially brown algae, and could therefore account for a potential carbon substrate for marine heterotrophic bacteria [42]. Our study showed that *V. diabolicus* could produce diabolican in the sole presence of mannitol as the carbon source, but this substrate was less efficient than glucose. In this case, the combination of different substrates, e.g., glucose and mannitol, could advantageously be further considered.

The effect of nitrogen sources on EPS production was also tested. Pomeroy (1974) indicated that organic nitrogen is usually preferred by heterotrophic bacteria as their role in the decomposition and mineralization of dissolved particulate organic nitrogen is important [43]. Romero Soto et al. studied the effect of carbon and nitrogen sources on the co-production of EPS and polyhydroxyalkanoate (PHA) in a moderately halophilic bacterium, *Yangia* sp. ND199 [32]. The highest EPS production was obtained with sucrose and fructose, while yeast extract and ammonium chloride were the most suitable nitrogen sources for cell growth and production of both polymers, over other inorganic sources such as ammonium sulfate, ammonium nitrate, and monosodium glutamate. On the other hand, MW was 24 times higher with yeast extract than with ammonium chloride [32]. Two *Lactobacillus rhamnosus* strains reached higher EPS yields without any nitrogenous substrate, while the production by a third strain was better in the presence of yeast extract [18]. This demonstrates that the production efficiency is species dependent, or even strain dependent. 

The intrinsic reason for the influence of various parameters in the cultivation process needs to be further studied, but they can be good tools for mitigating yield and MW in industrial processes and to ensure consistent EPS structures with the same proven biological properties.

Although nitrogen is the main substrate for cell growth and protein biosynthesis, nitrogen in a culture medium influences polysaccharide production by microorganisms. An excess of it may inhibit EPS production [44]. Sengupta et al. suggested that production optimization should rather be based on the C/N ratio of the medium [44]. Indeed, nitrogen limitation results in microbial growth limitation, and the carbon is more available for EPS synthesis [21]. In the case of *Yangia* sp., which produces both EPS and PHA, a slightly higher EPS yield was obtained with 20 g L^−1^ glucose using 2 g L^−1^ ammonium chloride as the nitrogen source (C/N of 10–16) instead of yeast extract (C/N of 30–35). This latter is more favorable to PHA production [32]. In *Haloferax mediterranei*, C/N ratios of 35 and 5 have been reported to increase PHA and EPS yields, respectively [45].

Other strategies could be envisaged to optimize diabolican production. A better knowledge of the metabolic pathways upon the cultivation of *V. diabolicus* with carbon in excess might be required to avoid channelizing the carbon substrate into multiple by-products such as acetic acid, which was identified in some *Vibrio* sp. [34,46]. In addition, since bacterial growth and EPS production are competing pathways in the bacterial strain, decoupling the biomass accumulation and the EPS production could be crucial for guaranteeing satisfactory yields in industrial processes [47]. Since cell membrane synthesis upon cell growth and EPS production are competitive in sharing UDP-nucleotide sugar precursors, changing UDP-sugars balance either by in-cell overexpression of the biosynthesis enzymes or by exogenous addition of sugars could mitigate the final EPS MW [22].

Enhanced EPS production yield with specific structural features could also be achieved by genome engineering. EPS biosynthesis is determined by genetics, which may undergo tight regulation [44,48,49]. However, although genomic sequencing of marine bacteria can allow the identification of the gene cluster involved in EPS biosynthesis, genome engineering to improve marine EPS production still need intense research.

## Figures and Tables

**Table 1:** Attributes of the CCD. Glucose or mannitol was the carbon source.

Medium Number	CCD Point	Ammonium Acetate (mM)	Carbon Source (g L^−1^)
1	Central-a	60	37.5
2	Central-b	60	37.5
3	Central-c	60	37.5
4	Cube01	20	15
5	Cube02	100	15
6	Cube03	100	60
7	Cube04	20	60
8	StarPointH	60	69.32
9	StarPointL	3.43	37.5
10	StarPointR	116.57	37.5
11	StarPointB	60	5.68

**Table 2:** Predicted or experimental yield and MW obtained for six ammonium acetate (AcA) and glucose (Glc) concentration combinations.

	AcA	Glc	Predicted Yield	Experimental Yield	Predicted MW	Experimental MW
	(mM)	(g L^−1^)	(mg L^−1^)	(mg L^−1^)	(g mol^−1^)	(g mol^−1^)
Opt.µ	3.4	5.7	208.6		1.74 × 10^6^	
Opt.MW	101.9	69.3	513.7		2.32 × 10^6^	
Opt.Yield	116.6	69.3	563.1		2.31 × 10^6^	
B1	100	60	450.1	497.3	2.23 × 10^6^	9.63 × 10^5^
B2	100	100	705.2	896.5	2.45 × 10^6^	5.37 × 10^5^
Zobell		30		588.9		8.21 × 10^5^

## Data Availability

Not applicable.

## Supplementary Materials

The following are available online at https://www.mdpi.com/article/10.3390/polym14101994/s1, Supplementary Data S1: Osidic composition of the EPS produced upon the CCD conditions, Supplementary data S2: Statistics from the CCD analyses with Statgraphics software.
