# Supplementary material for: Influence of the Carbon and Nitrogen Sources on Diabolican Production by the Marine Vibrio diabolicus Strain CNCM I-1629
Source: Polymers (Basel). 2022 May 13;14(10):1994. doi: 10.3390/polym14101994 (PMC9145141; doi:10.3390/polym14101994)

Supplementary data S1 : Osidic composition of the EPS produced upon the CCD conditions

On culture medium of the CCD Ammonium acetate – Glucose

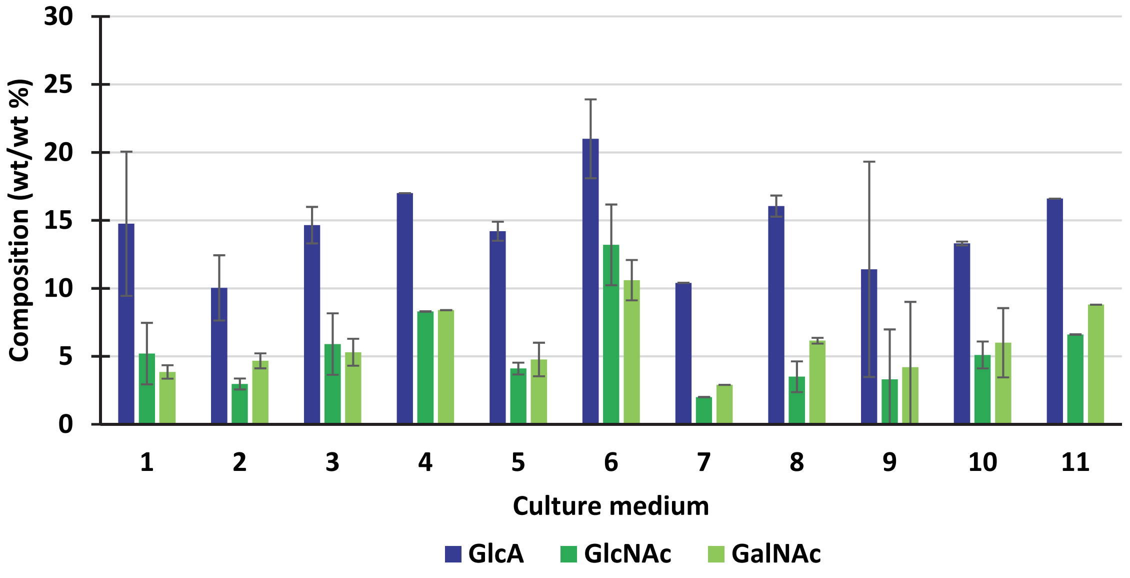

On culture medium of the CCD Ammonium acetate – Mannitol

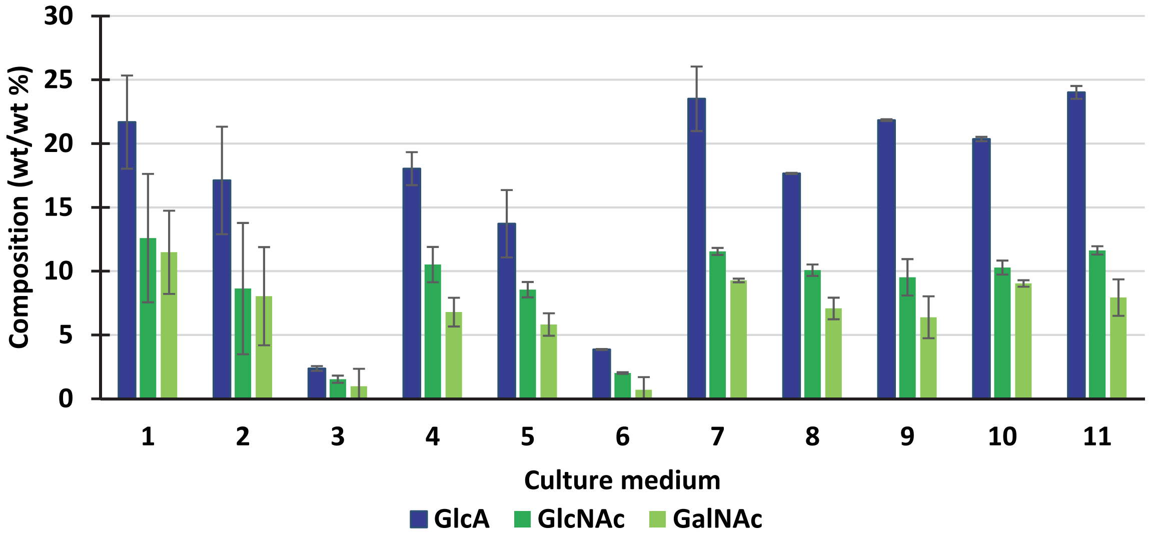

Supplement: Supplementary file 1 [file polymers-14-01994-s001.zip › Supplementary data S1.pdf]
