# Supplementary material for: Influence of the Carbon and Nitrogen Sources on Diabolican Production by the Marine Vibrio diabolicus Strain CNCM I-1629
Source: Polymers (Basel). 2022 May 13;14(10):1994. doi: 10.3390/polym14101994 (PMC9145141; doi:10.3390/polym14101994)
Supplement: Supplementary file 1 [file polymers-14-01994-s001.zip › Supplementary data S2.pdf]

Supplementary data S2 : Statistics from the CCD analyses with Statgraphics software

(ANOVA table, Model coefficients, Residual plot and Optimum value)

Ammonium acetate - Glucose : Growth rate (µmax, min-1)

Analysis of Variance

Source	*Sum of Squares*	*Df*	*Mean Square*	*F-Ratio*	*P-Value*
A:Ammonium acetate (mM)	0.000303606	1	0.000303606	500.36	0.0000
B:Glucose (g L-1)	0.0000440626	1	0.0000440626	72.62	0.0004
AA	1.52104E-7	1	1.52104E-7	0.25	0.6379
AB	5.57172E-10	1	5.57172E-10	0.00	0.9770
BB	4.24029E-8	1	4.24029E-8	0.07	0.8021
Total error	0.0000030339	5	6.0678E-7		
Total (corr.)	0.000350864	10			

R-squared = 99.1353 percent
R-squared (adjusted for d.f.) = 98.2706 percent
Standard Error of Est. = 0.000778961
Mean absolute error = 0.000440966
Durbin-Watson statistic = 1.29846 (P=0.3557)
Lag 1 residual autocorrelation = 0.271903

Regression coeffs

Coefficient	*Estimate*
constant	0.0366905
A:Ammonium acetate (mM)	-0.000166811
B:Glucose (g L-1)	-0.00011793
AA	1.02574E-7
AB	1.31136E-8
BB	1.71168E-7

Residual plot

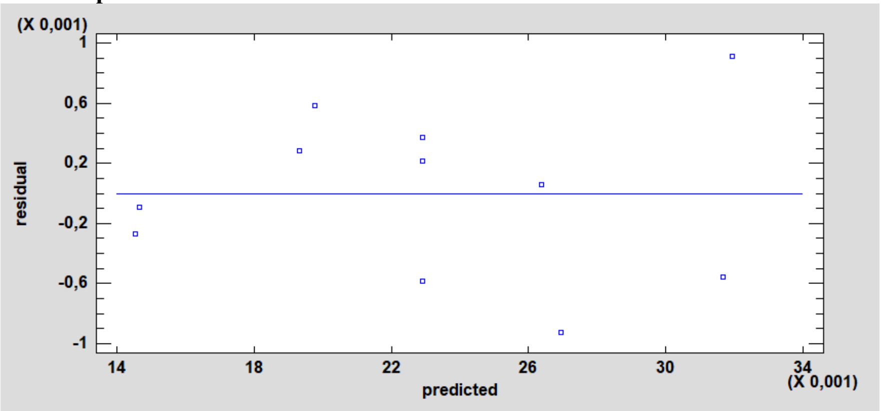

Optimum µ value = 0.0354 min^{-1} for 3.4 mM ammonium acetate and 5.7 g L^{-1} glucose

Ammonium acetate - Mannitol : Growth rate (µmax, min-1)

Analysis of Variance

Source	*Sum of Squares*	*Df*	*Mean Square*	*F-Ratio*	*P-Value*
A:Ammonium acetate (mM)	0.000323682	1	0.000323682	736.41	0.0000
B:Mannitol (g L-1)	8.54021E-7	1	8.54021E-7	1.94	0.2221
AA	4.15457E-8	1	4.15457E-8	0.09	0.7709
AB	1.62369E-7	1	1.62369E-7	0.37	0.5699
BB	5.39568E-7	1	5.39568E-7	1.23	0.3183
Total error	0.00000219771	5	4.39543E-7		
Total (corr.)	0.000327629	10			

R-squared = 99.3292 percent
R-squared (adjusted for d.f.) = 98.6584 percent
Standard Error of Est. = 0.00066298
Mean absolute error = 0.000421463
Durbin-Watson statistic = 0.988954 (P=0.1595)
Lag 1 residual autocorrelation = 0.393357

Regression coeffs

Coefficient	*Estimate*
constant	0.0373466
A:Ammonium acetate (mM)	-0.000160983
B:Mannitol (g L-1)	-0.000073747
AA	-5.36079E-8
AB	2.23861E-7
BB	6.10587E-7

Residual plot

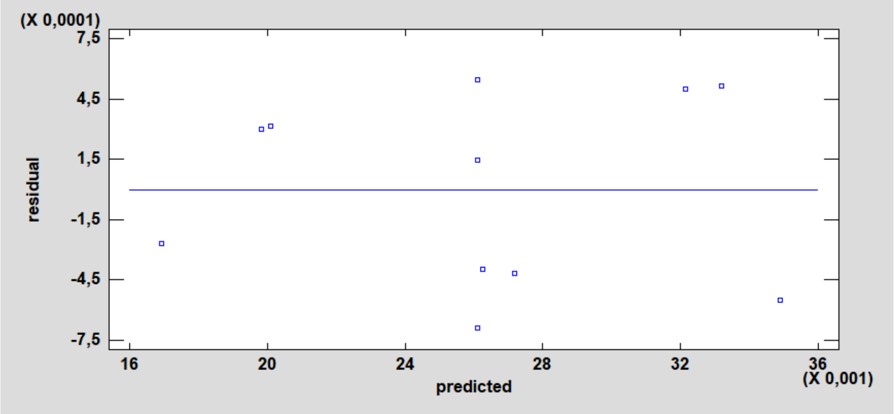

Optimum µ value = 0.0364 min^{-1} for 3.4 mM ammonium acetate and 5.7 g L^{-1} mannitol

Ammonium acetate - Glucose : Molecular Weight (MW, g L-1)

Analysis of Variance

Source	*Sum of Squares*	*Df*	*Mean Square*	*F-Ratio*	*P-Value*
A:Ammonium acetate (mM)	4.35019E10	1	4.35019E10	0.57	0.4833
B:Glucose (g L-1)	6.09512E11	1	6.09512E11	8.02	0.0366
AA	1.06442E9	1	1.06442E9	0.01	0.9104
AB	2.1025E10	1	2.1025E10	0.28	0.6213
BB	2.06554E10	1	2.06554E10	0.27	0.6243
Total error	3.79828E11	5	7.59656E10		
Total (corr.)	1.07462E12	10			

R-squared = 64.6548 percent
R-squared (adjusted for d.f.) = 29.3096 percent
Standard Error of Est. = 275619.
Mean absolute error = 145621.
Durbin-Watson statistic = 0.909832 (P=0.1209)
Lag 1 residual autocorrelation = 0.218778

Regression coeffs.

Coefficient	*Estimate*
constant	1.66527E6
A:Ammonium acetate (mM)	-3834.67
B:Glucose (g L-1)	16394.3
AA	-8.58073
AB	80.5556
BB	-119.465

Residual plot

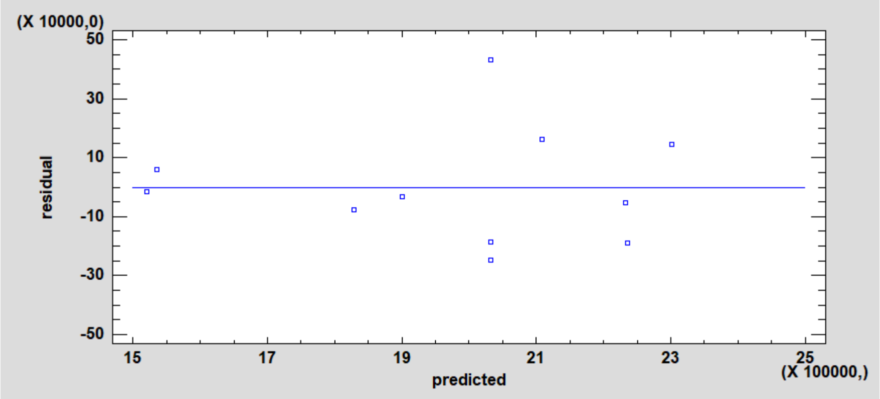

Optimum MW value = 2.316820 E6 g L^{-1} for 101.9 mM ammonium acetate and 69.3 g L^{-1} glucose

Ammonium acetate - Mannitol : Molecular Weight (MW, g L-1)

Analysis of Variance

Source	*Sum of Squares*	*Df*	*Mean Square*	*F-Ratio*	*P-Value*
A:Ammonium acetate (mM)	1.85772E12	1	1.85772E12	3.64	0.1146
B:Mannitol (g L-1)	1.23221E12	1	1.23221E12	2.42	0.1808
AA	3.56979E11	1	3.56979E11	0.70	0.4410
AB	9.44333E10	1	9.44333E10	0.19	0.6849
BB	1.66016E12	1	1.66016E12	3.25	0.1311
Total error	2.5502E12	5	5.1004E11		
Total (corr.)	7.44701E12	10			

R-squared = 65.7553 percent
R-squared (adjusted for d.f.) = 31.5107 percent
Standard Error of Est. = 714171.
Mean absolute error = 395566.
Durbin-Watson statistic = 2.84748 (P=0.9935)
Lag 1 residual autocorrelation = -0.489777

Regression coeffs.

**Coefficient**	**Estimate**
**constant**	**-30236.5**
**A:Ammonium acetate (mM)**	**13211.8**
**B:Mannitol (g L-1)**	**108013.**
**AA**	**-157.14**
**AB**	**-170.722**
**BB**	**-1071.02**

Residual plot

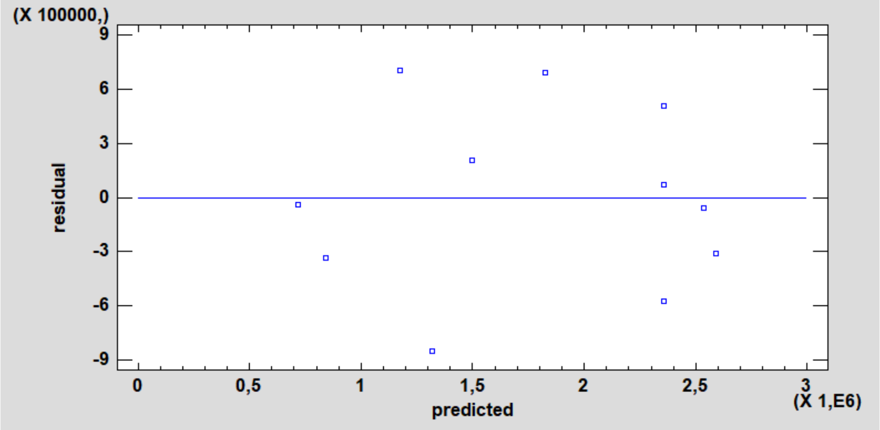

Optimum MW value = 2.72828 E6 g L^{-1} for 15.3 mM ammonium acetate and 49.2 g L^{-1} mannitol

Ammonium acetate - Glucose : Yield (mg L-1)

Analysis of Variance

Source	*Sum of Squares*	*Df*	*Mean Square*	*F-Ratio*	*P-Value*
A:Ammonium acetate (mM)	56330.0	1	56330.0	26.99	0.0035
B:Glucose (g L-1)	13716.5	1	13716.5	6.57	0.0504
AA	4436.59	1	4436.59	2.13	0.2046
AB	28556.1	1	28556.1	13.68	0.0140
BB	121.881	1	121.881	0.06	0.8186
Total error	10434.9	5	2086.97		
Total (corr.)	114501.	10			

R-squared = 90.8867 percent
R-squared (adjusted for d.f.) = 81.7734 percent
Standard Error of Est. = 45.6834
Mean absolute error = 27.9143
Durbin-Watson statistic = 1.37764 (P=0.4130)
Lag 1 residual autocorrelation = 0.195928

Regression coeffs.

Coefficient	*Estimate*
constant	229.89
A:Ammonium acetate (mM)	0.679476
B:Glucose (g L-1)	-4.48079
AA	-0.0175184
AB	0.0938808
BB	0.00917682

Residual plot

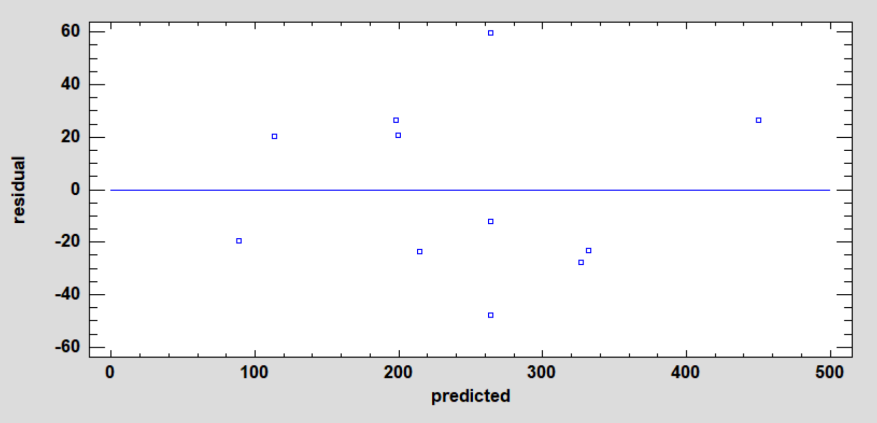

Optimum yield value = 563.1 g L-1 for 116.6 mM ammonium acetate and 69.3 g L^{-1} glucose

Ammonium acetate - Mannitol : Yield (mg L-1)

Analysis of Variance

Source	*Sum of Squares*	*Df*	*Mean Square*	*F-Ratio*	*P-Value*
A:Ammonium acetate (mM)	49366.4	1	49366.4	8.15	0.0356
B:Mannitol (g L-1)	12202.1	1	12202.1	2.02	0.2150
AA	3634.73	1	3634.73	0.60	0.4735
AB	503.161	1	503.161	0.08	0.7847
BB	2324.01	1	2324.01	0.38	0.5627
Total error	30275.5	5	6055.1		
Total (corr.)	100742.	10			

R-squared = 69.9475 percent
R-squared (adjusted for d.f.) = 39.895 percent
Standard Error of Est. = 77.8145
Mean absolute error = 45.9084
Durbin-Watson statistic = 1.39783 (P=0.4278)
Lag 1 residual autocorrelation = 0.228255

Regression coeffs.

Coefficient	*Estimate*
constant	137.743
A:Ammonium acetate (mM)	-0.406219
B:Mannitol (g L-1)	0.521944
AA	0.0158563
AB	0.0124618
BB	-0.0400722

Residual plot

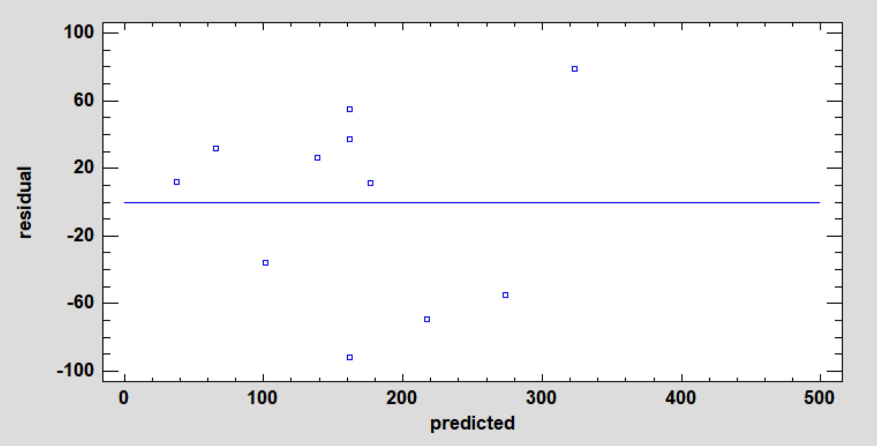

Optimum yield value = 330.2 g L-1 for 116.6 mM ammonium acetate and 24.6 g L^{-1} mannitol
